# Supplementary figures and images for: AleRax: a tool for gene and species tree co-estimation and reconciliation under a probabilistic model of gene duplication, transfer, and loss
Source: Bioinformatics. 2024 Mar 21;40(4):btae162. doi: 10.1093/bioinformatics/btae162 (PMC10990685; doi:10.1093/bioinformatics/btae162)

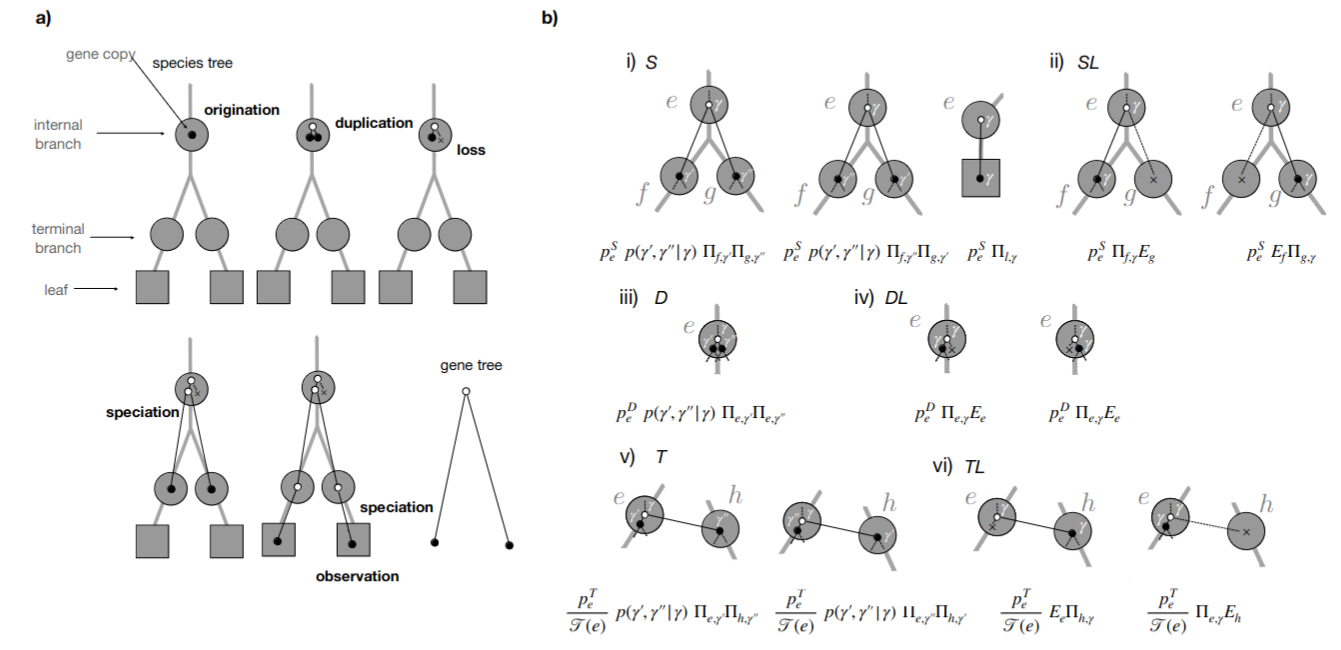

Supplement: btae162_Supplementary_Data [file btae162_supplementary_data.zip › figure1_supp.png]
